# Insight into cannabis use disorder in people with psychotic disorders

**DOI:** 10.1192/bjo.2026.12064

**Published:** 2026-08-25

**Authors:** Edward Chesney, Millie Lloyd, Sebastien Brodeur, Arsime Demjaha, Lisa Conlan, Ikram Slimani, Ayşe Doğa Lamper, Ananya Sarma, Will Lawn, Dominic Oliver, Tom P. Freeman, Robin Murray, Marta Di Forti, Amir Englund, John Strang, Philip McGuire

**Affiliations:** Addictions Department, Institute of Psychiatry, Psychology and Neuroscience, King’s College London, London, UK; South London and Maudsley NHS Foundation Trust, London, UK; Department of Psychology, Royal Holloway, University of London, London, UK; Department of Psychiatry and Neuroscience, Université Laval, Québec City, Québec, Canada; CERVO Brain Research Centre, Québec City, Québec, Canada; Department of Psychosis Studies, Institute of Psychiatry, Psychology and Neuroscience, King’s College London, London, UK; Department of Psychology, Institute of Psychiatry, Psychology and Neuroscience, King’s College London, London, UK; Department of Psychiatry, University of Oxford, Warneford Hospital, Oxford, UK; Centre for Drug Use Research, University of Bath, Bath, UK; Social, Genetic & Developmental Psychiatry Centre, Institute of Psychiatry, Psychology and Neuroscience, King’s College London, London, UK; Centre for Neuroimaging Sciences, Institute of Psychiatry, Psychology and Neuroscience, King’s College London, London, UK; NIHR Oxford Health Biomedical Research Centre, Oxford, UK

**Keywords:** Cannabis use disorder, cannabis dependence, cannabis, schizophrenia, psychosis

## Abstract

**Background:**

Cannabis use is common in people with psychosis and is associated with poor outcomes.

**Aims:**

To assess insight into cannabis use disorder, perceptions about how cannabis use influences mental health and the desire to change cannabis use among people with psychotic disorders.

**Method:**

Adults with a psychotic disorder and comorbid cannabis use disorder were asked about the impact of cannabis use on their illness and their desire to change cannabis use. Cannabis use disorder was determined using DSM-5 criteria. Participants completed the Substance Use Awareness and Insight Scale.

**Results:**

Sixty-two participants (mean age: 42 years; 95% male; 65% Black ethnicity; 85% severe DSM-5 cannabis use disorder) completed the study. The mean total Substance Use Awareness and Insight Scale score was 4.8 (s.d.: 2.1), with subdomain scores of 3.7 (s.d.: 2.8) for general illness awareness, 6.2 (s.d.: 2.8) for symptom attribution, 3.5 (s.d.: 2.9) for awareness of need for treatment and 5.6 (s.d.: 3.8) for awareness of negative consequences. Fifty-eight per cent said that cannabis use made their mental health ‘better’, whereas only 15% said it made it ‘worse’; 27% reported that cannabis made their psychotic symptoms ‘better’, and 21% said it made them ‘worse’. Nevertheless, more than half the sample (53%) said they wanted to reduce or stop using cannabis.

**Conclusions:**

Many people with psychotic disorders have impaired insight into cannabis use disorder or believe that cannabis improves their mental health; these factors may contribute to persistent use in this population. Interventions that improve insight may help to address ambivalence and support those already wishing to reduce use.

Cannabis use is very common among people with schizophrenia spectrum and related psychotic disorders, with up to 65% reporting lifetime use.^1^ A meta-analysis estimated that 36% of those with psychosis and 26% of those with schizophrenia met criteria for cannabis use disorder (CUD), defined as a problematic pattern of use with clinically significant impairment or distress.^2^ Among people with psychotic disorders, cannabis use is associated with more severe psychotic symptoms, lower treatment adherence, and increased risks of relapse, hospital admission and violence.^1,3–5^ It is therefore an important clinical issue for many people with psychotic disorders. Moreover, reducing cannabis use in this group is associated with better clinical outcomes.^3,4^

Clinicians routinely advise people with psychotic disorders to stop or at least reduce their cannabis use. Although some patients follow this advice, most continue to use cannabis. In one study, the proportion increased slightly from 65% at onset to 66% 1 year later.^6^ Evidence on longer-term patterns of use is mixed. One study found that cannabis use had decreased 3 years after the onset of psychosis,^7^ whereas another reported that it had increased after 2 years.^8^ However, both these studies found a decrease in use at 10 year follow-up. Evidence from the general population suggests that changes in cannabis use are shaped by individuals’ subjective experiences of the drug, with psychotic-like experiences increasing desire to quit and pleasurable effects reducing it.^9^ It is therefore important to investigate why people who have developed a psychotic disorder continue to use cannabis. In a systematic review of six qualitative studies, many patients rejected a causal link between cannabis use and psychosis onset, instead highlighting the roles of stress, social adversity, psychological factors and pre-existing vulnerability.^10^

In the general population, the total illness burden from CUD is considerably lower than that linked to alcohol or opioid dependence. However, among individuals with psychosis, cannabis use is associated with significantly greater harm than in individuals without psychosis. As a result, at a population level, the majority of harms attributable to cannabis use occur in people with psychosis.^11^ However, most people with psychosis do not seek clinical help for cannabis-related problems.^12^ This may be because cannabis can provide short-term relief for symptoms associated with psychosis, such as insomnia, anxiety, dysphoria and psychotic experiences.^13^ In addition, the majority of people who use cannabis do not think that it is addictive,^14^ despite evidence that about one in eight users develop dependence.^15^ There is also emerging evidence that cannabis withdrawal syndrome, which is observed in the majority of people with cannabis dependence on cessation of cannabis, can be associated with the onset of psychosis,^16^ as well as admission to psychiatric intensive care in psychosis populations.^17^ The perceived benefits of cannabis use, whether to relieve symptoms of psychotic illness, avoid withdrawal symptoms or for recreational purposes, may contribute to continued use despite harm.

Insight is a multidimensional construct that includes both clinical insight and cognitive insight.^18–20^ Clinical insight refers to awareness of having a disorder, awareness of its symptoms, recognition of the need for treatment and awareness of the consequences of the disorder.^19^ Cognitive insight refers to the capacity to reflect on and re-evaluate one’s interpretations and beliefs, including openness to corrective feedback and reduced overconfidence in one’s judgements.^18^ Although impaired insight is relevant to both psychosis and substance use, the respective characteristics and underlying mechanisms may differ. In individuals with schizophrenia, impaired clinical insight is common and has been understood using several explanatory models, including psychological, cognitive and/or neuropsychological, clinical and/or psychopathological, and neuroanatomical accounts.^19^ Neural correlates of impaired clinical insight have been studied relatively extensively in schizophrenia, with findings implicating frontoparietal and related self-referential networks.^21–23^ Impairments of insight in people with schizophrenia have a major influence on treatment adherence, prognosis and quality of life.^24^ Among individuals with substance use disorders, insight is also increasingly understood as multidimensional and not reducible to denial or minimisation alone. More broadly, the substance use disorder literature suggests that impaired insight may reflect disturbances in self-monitoring, metacognition, readiness for change, interoception and related self-awareness processes.^25^ Preliminary work has also begun to identify possible neural correlates of impaired illness awareness in substance use disorders, although this literature remains very limited.^26^

The aims of the present study were to assess, in people with psychotic disorders: (a) CUD clinical insight; (b) perceptions about how cannabis use influences mental health; and (c) desire to change cannabis use.

## Method

### Ethics

All procedures contributing to this work comply with the ethical standards of the relevant national and institutional committees on human experimentation and with the Helsinki Declaration of 1975, as revised in 2013. All procedures involving human participants and/or patients were approved by the Health and Social Care Research Ethics Committee A (HSC REC A-20/NI/0074) and were conducted in compliance with the principles of Good Clinical Practice. This study adheres to STROBE for cross-sectional studies (the checklist is provided in the Supplementary Material available at https://doi.org/10.1192/bjo.2026.12064).

### Design

This cross-sectional study was conducted at the National Institute for Health and Care Research (NIHR) Wellcome Trust Clinical Research Facility at King’s College Hospital, London, UK, between October 2021 and July 2023.

### Participants

Participants were recruited from south-east London via the South London and Maudsley NHS Foundation Trust. Participants were recruited from both out-patient clinics and in-patient wards. In-patients were invited to complete assessments at least 3 months after discharge from hospital. The inclusion criteria were: (a) diagnosis of a psychotic disorder (including psychosis spectrum [ICD-10: F20-29] and cannabis-induced psychotic disorder [F12.5]) or bipolar affective disorder (with psychotic symptoms) (F31); (b) diagnosis of DSM-5 CUD; (c) regular cannabis use (at least weekly); (d) self-reported cannabis use within the past 7 days; and (e) urine immunoassay test positive for cannabis (carboxy-THC).

### Procedure

Written informed consent was obtained from all participants. Demographic and clinical information was collected via interview and review of clinical records. Participants also completed self-report psychological scales and a semi-structured clinical interview. Participants were assessed for CUD by an experienced psychiatrist using DSM-5 criteria. A urine sample was collected to confirm recent use of cannabis.

### Measures

#### Substance Use Awareness and Insight Scale

The SAS is a seven-item self-report tool that assesses four domains of substance use insight: (a) general illness awareness, (b) symptom attribution, (c) awareness of the need for treatment and (d) awareness of the negative consequences of substance use.^27^ Items are scored on a ten-point Likert scale (0, strongly disagree; 10, strongly agree). Items 4 and 5 are reverse scored. A subtotal for each core domain is given, as well as an average total score, with higher scores indicating greater insight into substance use disorder. The scale was adapted here, replacing the term ‘substance’ with ‘cannabis’ for each item.

#### Psychosis symptoms and insight

Psychosis symptoms were assessed with the positive and negative subscales of the Positive and Negative Syndrome Scale (PANSS).^28^ To assess psychosis insight we used the ‘lack of judgement and insight’ item (G12) from the general psychopathology subscale. This is defined as ‘impaired awareness or understanding of one’s own psychiatric condition and life situation’ as evidenced ‘by failure to recognize past or present psychiatric illness or symptoms, denial of need for psychiatric hospitalization or treatment, decisions characterized by poor anticipation of consequences, and unrealistic short-term and long-range planning’. The item is scored on a seven-point scale (1, absent; 2, minimal; 3, mild; 4, moderate; 5, moderate severe; 6, severe; 7, extreme). All assessments were completed or reviewed by a single trained psychiatrist to minimise potential variability in administration.

#### DSM-5 criteria for CUD

To meet the criteria for CUD, an individual must have been using cannabis for a minimum of 1 year and present with two or more of 11 symptoms, accompanied by an impairment in functioning. Severity of CUD was categorised as mild (2–3 symptoms), moderate (4–5 symptoms) or severe (≥6 symptoms). All assessments involved an experienced psychiatrist.

#### Substance use history

Participants completed a modified version of the Cannabis Experience Questionnaire,^29^ providing information about their age at first use, frequency of use, number of joints per day, amount of cannabis use, spending on cannabis, preferred type of cannabis, tobacco co-use and other substance use. They also completed the Cannabis Use Disorder Identification Test – Revised (CUDIT-R), an eight-item self-report tool used to identify problematic or harmful cannabis use in the past 6 months.^30^ A total score of 8 or more indicates hazardous cannabis use, whereas a score of 12 or more indicates possible CUD. Participants also completed the Alcohol Use Disorders Identification Test,^31^ a ten-item self-report tool that identifies individuals with hazardous or harmful alcohol use in the past year. A total score of 8 or more indicates hazardous or harmful alcohol use, and scores of 20 or more suggest possible alcohol dependence.

#### Cannabis and Psychosis Impact Scale

This three-item questionnaire was developed for the present study. Participants were asked the following questions: (a) ‘Does cannabis affect your mental health?’ (worse, no difference, better); (b) ‘Does cannabis affect your psychotic symptoms?’ (worse, no difference, better); and (c) ‘Do you want to change your cannabis use?’ (use more, keep the same, use less).

### Statistical analysis

Means and standard deviations were used to determine 95% confidence intervals for SAS total and subscale scores. We compared these scores with those obtained from a previously recruited Canadian sample of people with harmful or dependent use of cannabis^27^ using a two-tailed *t*-test. This comparison sample had been recruited online, required a self-reported Drug Use Disorders Identification Test score ≥8, and included participants with and without psychiatric disorders. Kendall’s tau correlation analyses were performed to examine the relationship between SAS scores and psychosis insight. A logistic regression model was used to predict accurate self-diagnosis of CUD with the CUDIT-R according to the SAS general illness awareness domain. Kendall’s tau correlation analyses were performed to examine the relationships of (a) SAS scores and (b) psychosis insight with two clinical outcomes: number of hospital admissions and cannabis consumption (grams per day). Logistic regression was used to examine factors associated with desire to reduce or stop using cannabis. Predictor variables included age, gender, ethnicity, cannabis use (grams per day), years of use, CUDIT-R score and SAS scores. Variables that were statistically significant in univariate analyses were entered into a multivariable model. A threshold of *P* < 0.05 was used to establish significance. All analyses were completed in R version 4.3.1 for Mac (R Foundation for Statistical Computing, Vienna, Austria; https://www.r-project.org/).

## Results

Between October 2021 and July 2023, 273 individuals (83% male) were screened for inclusion. A greater proportion of men than women met the study criteria (37% *v*. 20%). Women were more often excluded as they denied current cannabis use (35% *v*. 26%). Sixty-two participants completed the study. Their demographic and clinical characteristics are reported in Table 1. Participants were predominantly male, of Black ethnicity and unemployed; the majority were diagnosed with schizophrenia (81%), with smaller numbers having diagnoses of schizoaffective disorder, bipolar disorder with psychotic symptoms, psychosis not otherwise specified and cannabis-induced psychosis. Most participants (85%) had severe CUD, defined as meeting at least 6 of 11 criteria. The majority (*n* = 36 [58%]) were daily cannabis users, with a mean use of 1.2 g (s.d.: 0.9) of cannabis per day and an average duration of use of 25 years. All participants smoked cannabis with tobacco, and most (82%) also used tobacco separately. A significant minority (*n* = 21; 34%) had used stimulants (i.e. cocaine, crack cocaine, amphetamine or methamphetamine) within the past year, including 15 (24%) who had a positive urine immunoassay test. Most were being treated with long-acting injectable antipsychotic medications. The average severity of psychosis symptoms on the PANSS positive and negative subscales was in the mild range (≤14).

**Table 1:** Demographic and clinical characteristics of the study population Table 1 long description.

Characteristics	Mean [s.d.] / *n* (%)
Age	
Years	42.2 [11.9]
Gender	
Male	59 (95.2)
Female	3 (4.8)
Ethnicity	
Black	40 (64.5)
White	18 (29.0)
Other	4 (6.5)
Education	
Secondary education	41 (66.1)
Vocational training	15 (24.2)
University	6 (9.7)
Employment	
Employed and/or in education	6 (9.7)
Unemployed	56 (90.3)
Marital status	
Single	49 (79.0)
In a relationship	8 (12.9)
Divorced or separated	5 (8.1)
Primary diagnosis	
Cannabis-induced psychotic disorder	1 (1.6)
Schizophrenia	50 (80.6)
Schizoaffective disorder	5 (8.1)
Psychosis not otherwise specified	3 (4.8)
Bipolar affective disorder (with psychotic symptoms)	3 (4.8)
Duration of illness	
Years	15.9 [11.4]
Psychiatric hospital admissions	
Past 10 years	2.7 [2.9]
Antipsychotic	
Long-acting injectable	45 (72.6)
Oral	17 (27.4)
Clozapine treatment	
Currently prescribed	5 (8.1)
Ever prescribed or recommended	12 (19.4)
Psychosis symptoms (PANSS)	
Positive symptoms	13.7 [5.3]
Negative symptoms	13.7 [6.7]
Remission (PANSS P1, P2, P3, N1, N4, N6 ≤3)	
Yes	23 (37.1)
History of violent crime	
Yes	36 (58.1)
Tobacco, alcohol and substance use^a^	
Tobacco use^b^	51 (82.3)
Alcohol use	49 (79.0)
Cocaine and/or amphetamine use	21 (33.9)
Opioid use	6 (9.7)
Synthetic cannabinoid use	3 (4.8)
AUDIT score	
Low risk	50 (80.6)
Increasing risk	9 (14.5)
Possible dependence	3 (4.8)
Cannabis use	
Age at first use, years	16.8 [6.1]
Duration of use, years	25.4 [12.9]
Days use per week	5.5 [1.9]
Joints per day	6.0 [4.6]
Grams per day	1.2 [0.93]
Spending (£) per day	9.4 [6.5]
Preferred type of cannabis	
High potency cannabis	54 (87.1)
Low potency	8 (12.9)
Preferred route of administration	
Smoking	62 (100)
Tobacco co-use	
Yes	62 (100)
DSM-5 cannabis use disorder	
Mild	3 (4.8)
Moderate	6 (9.7)
Severe	53 (85.5)
CUDIT-R	
Missing	4 (6.5)
Low risk	5 (8.1)
Hazardous use	13 (21.0)
Possible cannabis use disorder	40 (64.5)

PANSS, Positive and Negative Syndrome Scale; AUDIT, Alcohol Use Disorders Identification Test; CUDIT-R, Cannabis Use Disorder Identification Test – Revised.

a. Either self-reported use within the past 12 months or positive urine immunoassay.

b. Independent of cannabis use.

### Insight into CUD

**Fig. 1:**
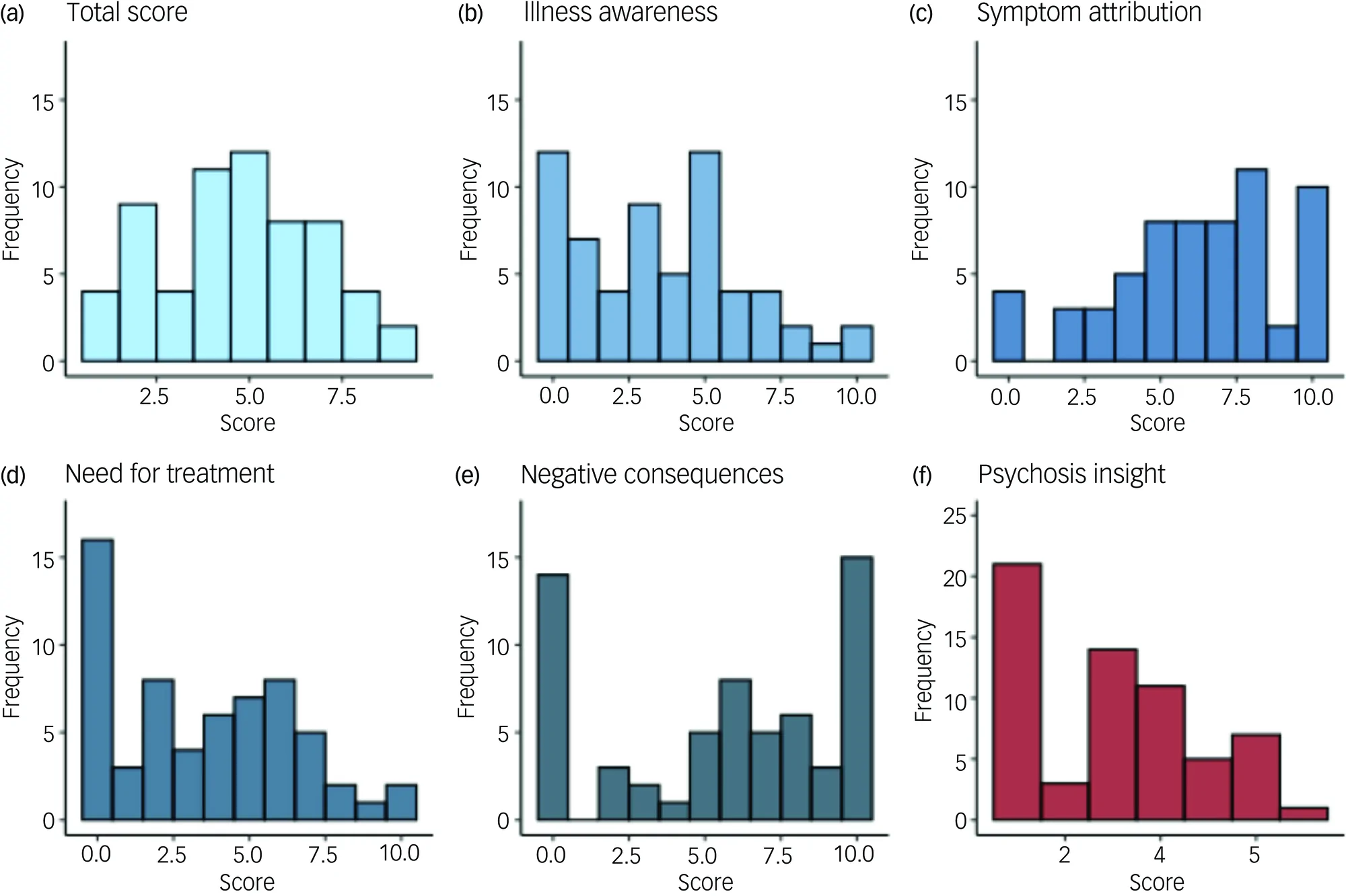
(a–e) Insight into cannabis use disorder as measured by SAS. (f) Insight into psychosis as measured by PANSS item G12.

The mean total SAS score was 4.8 (s.d.: 2.1), indicating a moderate overall level of substance use insight. The subdomain scores were 3.7 (s.d.: 2.8) for general illness awareness, 6.2 (s.d.: 2.8) for symptom attribution, 3.5 (s.d.: 2.9) for awareness of need for treatment and 5.6 (s.d.: 3.8) for awareness of negative consequences (Fig. 1(a–e)).

Compared with a Canadian sample of people with harmful or dependent use of cannabis,^27^ our sample had a higher mean total SAS score (4.8 [s.d.: 2.1] *v*. 3.5 [s.d.: 2.4]; *P* = 0.001). Scores on the general illness awareness subdomain were comparable across the two samples (3.7 [s.d.: 2.8] *v*. 3.3 [s.d.: 2.5]; *P* = 0.34), whereas our sample had higher scores for symptom attribution (6.2 [s.d.: 2.8] *v*. 4.8 [s.d.: 3.0]; *P* = 0.003), awareness of need for treatment (3.5 [s.d.: 2.9] *v*. 2.5 [s.d.: 2.4]; *P* = 0.02) and awareness of negative consequences (5.6 [s.d.: 3.8] *v*. 3.5 [s.d.: 2.4]; *P* < 0.0001).

### Psychosis insight and correlation with insight into CUD

The mean score on PANSS item G12 was 3.0 (s.d.: 1.8) (Fig. 1(f)). Twenty-four participants (39%) demonstrated no or minimal impairment in psychosis insight (G12 ≤ 2), 25 (40%) had mild to moderate impairments (G12: 3–4) and 13 (21%) had severe impairments (G12 ≥ 5).

There was a significant negative correlation of moderate magnitude between PANSS-G12 and SAS total score (tau = −0.26; *P* = 0.005), with greater severity of psychosis insight impairment associated with a lesser degree of insight into CUD. There was also a significant negative correlation of moderate size between PANSS-G12 and SAS general illness awareness (tau = −0.25; *P* = 0.01) and one of small size between PANSS-G12 and SAS symptom attribution (tau = −0.21; *P* = 0.04). The correlations between PANSS-G12 and other SAS subdomains were not statistically significant (awareness of need for treatment [tau = −0.15; *P* = 0.12] and awareness of negative consequences [tau = −0.08; *P* = 0.41]).

### CUD diagnosis and CUDIT-R self-assessment

All participants were assessed against DSM-5 criteria for CUD. In most (53; 85%), CUD was rated as severe (6–11 criteria). Six participants (10%) had moderate CUD (4–5 criteria), and three (5%) had mild CUD (meeting 2–3 criteria). Fifty-eight participants also completed the CUDIT-R scale. The mean score was 15.2 (s.d.: 5.9). Five (9%) participants scored below 8 (low risk), 13 (22%) scored between 8 and 11 (hazardous use), and 40 (69%) had a score ≥12 (indicating possible CUD), including 39 of the 52 with severe CUD (75%). Among participants who had a score on the general illness awareness domain of the SAS of 4 or above, 26 of 28 (93%) classified themselves as being at risk of CUD. For those with a score below 4, 14 of 30 (47%) classified themselves as being at risk of CUD (odds ratio = 14.9 [95% CI: 3.6 to 103.0], *P* = 0.001).

### Insight and clinical outcomes

SAS total score had a small-to-moderate association with severity of CUD, whether measured by DSM-5 CUD symptoms (tau = 0.24; *P* = 0.01) or CUDIT-R total score (tau = 0.38; *P* < 0.0001). SAS total score was not associated with amount of cannabis use (grams per day) (tau = 0.10; *P* = 0.29) or number of psychiatric admissions over the past 10 years (tau = −0.04; *P* = 0.67).

Psychosis insight (G12 on PANSS) had a statistically significant association of small–moderate magnitude with number of admissions (tau = 0.23; *P* = 0.02), with greater impairment associated with more admissions. It was also associated with severity of PANSS positive symptoms (tau = 0.24; *P* = 0.01) but not that of PANSS negative symptoms (tau = 0.09; *P* = 0.37). Psychosis insight was not associated with amount of cannabis use (tau = 0.04; *P* = 0.66).

### Perceived relationship between cannabis use and mental health

When asked how cannabis affected their overall mental health, among 61 responders, 36 (58%) participants reported that it made it ‘better’, 17 (27%) said it made ‘no difference’ and nine (15%) said it made it ‘worse’. When asked how cannabis affected their psychosis symptoms, 17 (27%) participants reported that it made it ‘better’, 31 (50%) said it made ‘no difference’ and 13 (21%) said it made it ‘worse’. The most commonly cited benefits were calming and/or relaxing effects (*n* = 19), sleep (*n* = 5), mood (*n* = 4), hallucinations (*n* = 4), appetite (*n* = 3), agitation and irritability (*n* = 3) and motivation (*n* = 3). The most commonly cited negative effects were paranoia (*n* = 8), anxiety (*n* = 3), mood (*n* = 3) and motivation (*n* = 3).

To examine the role of residual symptoms, we assessed correlations between psychotic symptoms and amount of cannabis use (grams per day). There was no association with PANSS positive symptoms (tau = −0.02, *P* = 0.80). Negative symptoms showed a small but statistically significant negative correlation (tau = −0.20, *P* = 0.04).

### Desire to change cannabis use

**Fig. 2:**
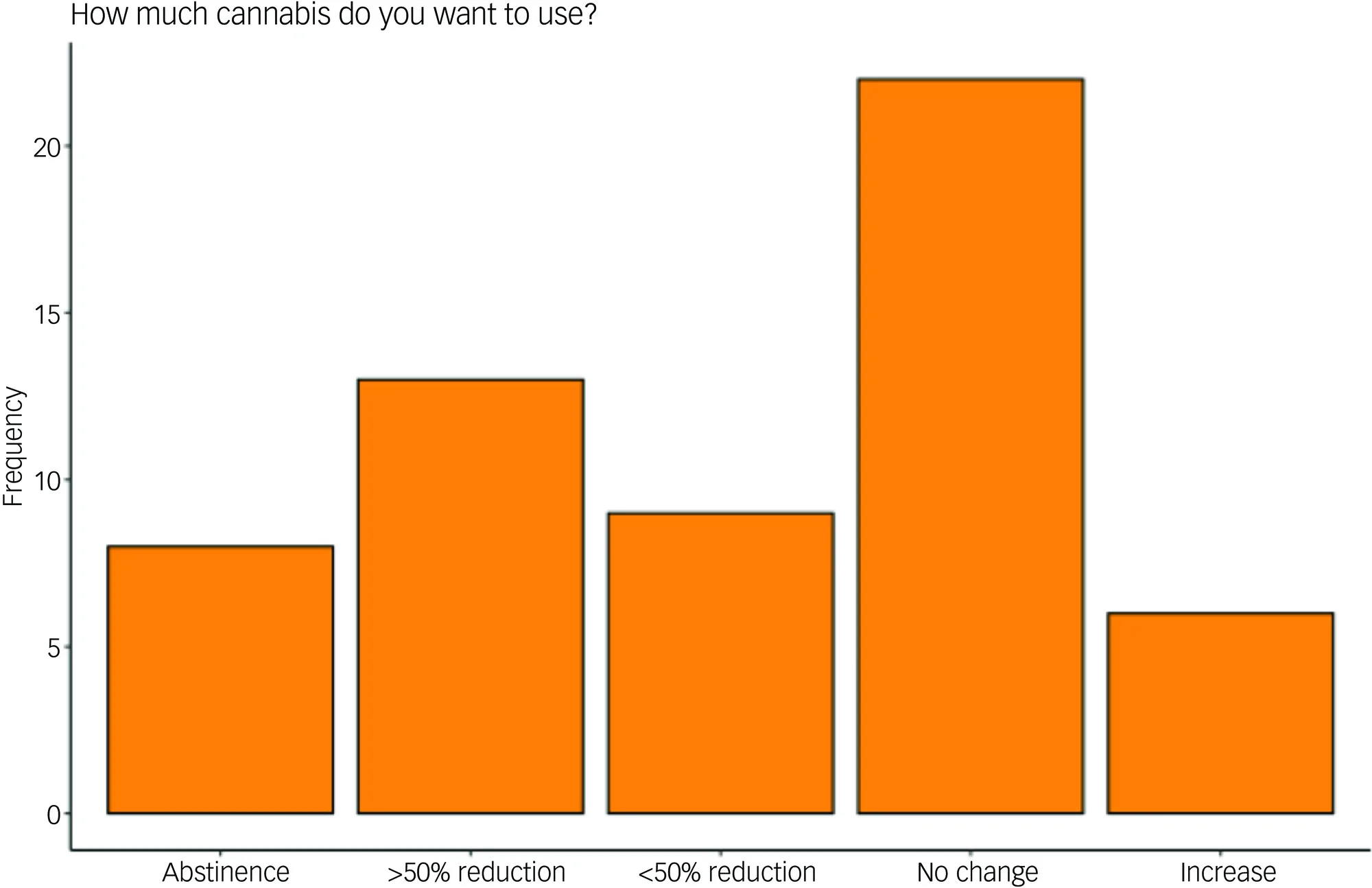
Target cannabis use (assessed in either grams per week or £ per week) relative to current use.

When asked whether they wanted to change the amount of cannabis they were using, 33 participants (53%) said they wanted to use less, 22 (36%) said they wanted to keep using the same amount and 6 (10%) said they wanted to use more. Of the participants who wanted to cut down their use, 8 (27%) said they wanted to abstain from cannabis completely, and 22 (73%) wanted to reduce their use (Fig. 2).

Wanting to reduce or stop cannabis use was associated with greater daily cannabis consumption, higher overall SAS scores, and elevated scores on the general illness awareness, awareness of negative consequences and awareness of need for treatment subscales (Table 2). Demographic factors (age, gender, ethnicity), years of cannabis use, PANSS psychosis symptoms, PANSS psychosis insight, CUDIT-R score and SAS symptom attribution subscale score were not associated with wanting to reduce or stop cannabis use. In the multivariable model, greater daily cannabis consumption (odds ratio = 4.5, 95% CI: 1.6–19.7; *P* = 0.02) and higher scores on the awareness of need for treatment subscale (odds ratio = 1.6, 95% CI: 1.2–2.3; *P* = 0.007) remained as significant predictors. Participants who wished to cut down or quit used an average of 1.5 grams per day (s.d. = 1.1), compared with 0.92 grams per day (s.d. = 0.53) among those who did not (*P* = 0.005). Forty-eight participants were asked whether they thought they were addicted to cannabis, and 36 (75%) agreed.

**Table 2:** Univariate logistic regression of factors which predict desire to reduce or stop cannabis use Table 2 long description.

Factor	Odds ratio (95% CI)	*P*-value
Age	0.99 (0.94 to 1.03)	0.44
Gender	1.74 (0.16 to 38.7)	0.66
Ethnicity (White)	0.59 (0.19 to 1.81)	0.36
Ethnicity (other)	1.48 (0.13 to 33.4)	0.76
Cannabis use (grams per day)	2.97 (1.36 to 8.44)	0.018
Years of use	0.99 (0.95 to 1.03)	0.75
CUDIT-R score	1.07 (0.98 to 1.18)	0.15
PANSS positive subscale	1.02 (0.93 to 1.13)	0.64
PANSS negative subscale	0.95 (0.87 to 1.02)	0.19
Psychosis insight (PANSS-G12)	0.83 (0.62 to 1.11)	0.22
SAS total score	1.76 (1.30 to 2.53)	0.0007
SAS general illness awareness	1.35 (1.10 to 1.70)	0.007
SAS symptom attribution	1.06 (0.89 to 1.28)	0.52
SAS awareness of negative consequences	1.20 (1.04 to 1.40)	0.014
SAS awareness of need for treatment	1.55 (1.24 to 2.02)	0.0003

CUDIT-R, Cannabis Use Disorder Identification Test–Revised; PANSS, Positive and Negative Syndrome Scale; SAS, Substance Use Awareness and Insight Scale.

## Discussion

This study aimed to assess CUD clinical insight, perceptions about how cannabis use influences mental health and desire to change cannabis use in people with psychotic disorders. Our main finding was that many people with schizophrenia and related psychotic disorders and problematic cannabis use did not recognise the negative impacts of their cannabis use. Nevertheless, half of the sample said they wanted to use less cannabis, suggesting that even if insight is impaired, interventions to reduce cannabis use have the potential to be effective.^32^

Our sample had a higher mean total SAS score, higher scores for symptom attribution, awareness of the need for treatment and awareness of negative consequences, and a similar score for general illness awareness compared with those reported by a study involving individuals from the Canadian general population with harmful or dependent use of cannabis.^27^ However, the samples in the two studies were quite different: our sample comprised people with psychotic disorders, whereas 46% of the Canadian sample reported a diagnosis of depression, anxiety or another mental disorder; in addition, our sample had a much higher proportion of men (95% *v*. 54%). Nevertheless, people with psychosis may be more vulnerable to the harms of cannabis^11^ and have more contact with clinicians who are likely to highlight the harms of their cannabis use;^32^ these factors may have influenced their perceptions and could partly explain why their scores on the SAS were higher.

The majority of participants (85%) in our study met DSM-5 criteria for severe CUD, yet a substantial minority did not recognise the negative impact of their use or feel that they needed treatment. This may partly explain why many people with psychosis continue to use cannabis after illness onset despite clinical advice to stop. For some individuals, cannabis may be perceived as providing short-term relief from distressing symptoms or stressful life events; however, the cross-sectional design of our study precluded inference about the direction of this relationship. In our analyses, participants with more severe negative symptoms used less cannabis, a pattern that is not readily explained by a self-medication hypothesis. Another relevant factor is cannabis withdrawal syndrome, which can cause unpleasant symptoms in heavy users if they stop using cannabis. Over time, the contrast between these immediate experiences and standard clinical explanations of the harms of cannabis may reduce the likelihood that patients perceive their drug use as harmful.

Participants’ subjective beliefs about cannabis use were mixed: although only a minority believed that cannabis worsened their overall mental health (15%) or psychotic symptoms (21%), three-quarters thought they were addicted to cannabis, and half expressed a desire to reduce or stop their use. Higher cannabis use and greater insight into CUD were associated with a desire to reduce use. These results suggest that many people with psychotic disorders are open to change, even if they do not believe that cannabis use is harming their mental health. One possible explanation for this is the cost of their habit: our participants reported that they spent an average of £66 per week (i.e. £3400 per year) on cannabis, an amount that is likely to account for a significant proportion of their disposable income. An alternative explanation is that although some patients with psychosis experience short-term benefits (e.g. symptom relief) from cannabis use, they may also appreciate that it can be damaging in the longer term, similar to cigarette smoking. Most current tobacco smokers are aware of the risks and would like to stop.^33^

Participants with better psychosis insight (assessed via PANSS interview) also tended to have better insight into their CUD, particularly illness awareness. However, this correlation was only modest (tau = −0.26), and it was not evident across all CUD clinical insight domains. A plausible explanation for the relatively limited strength of the correlation is that insight is domain-specific, and that insight into psychosis and insight into substance use disorder may rely on distinct cognitive, motivational and social mechanisms. Clinically, this may help to explain why some patients acknowledge having a psychotic disorder yet do not recognise their cannabis use as problematic, whereas others may accept that cannabis is harmful without recognising their psychotic illness. This distinction is important, because interventions may need to target these domains separately. For example, insight into CUD may be influenced by an individual’s perceived utility of cannabis, including symptom relief, pleasure and avoidance of withdrawal,^34^ whereas insight into psychosis may be shaped by several partially overlapping factors, including psychopathology, neurocognitive impairment, and broader psychological and neurobiological mechanisms.^19,20,34^ Consistent with this, we also found that greater CUD severity was associated with better CUD insight, supporting the view that insight may be shaped differently in psychotic and addictive disorders.

The best-established intervention for improving insight into CUD and readiness to change is motivational interviewing. A meta-analysis of 19 trials demonstrated that this intervention can reduce both the frequency and quantity of cannabis use.^35^ However, two trials conducted in people with psychotic disorders reported minimal benefit.^36,37^ Non-invasive neuromodulation approaches have also been studied: transcranial direct current stimulation has been used to enhance insight in psychosis, with a meta-analysis of 19 trials reporting positive effects.^38^ Recently, a small clinical trial evaluated repetitive transcranial magnetic stimulation as an adjunct to motivational interviewing in people with CUD, targeting craving reduction, and reported encouraging preliminary findings.^39^

The CUDIT-R showed relatively limited sensitivity as a screening tool in our sample, with one-third (33%) scoring below the threshold of 12. Notably, higher general illness awareness on the SAS was strongly associated with scoring above this threshold, underscoring the role of insight in self-reported screening tools. These findings are consistent with those of previous research in the general population, in which a CUDIT-R score ≥12 yielded a sensitivity of 69%,^40^ compared with 91% in treatment-seeking samples.^30^

To our knowledge, this is the first study to use the SAS scale in a psychosis spectrum population. The sample consisted of a patient group that is unlikely to receive specific interventions to help them to reduce their cannabis use, despite experiencing significant harm from cannabis.^12^ The mean age of participants in our study was 42 years, and most had a diagnosis of chronic schizophrenia or schizoaffective disorder. As a proportion of people who develop psychosis stop using cannabis around the time of illness onset,^41^ it may be worth replicating this study in a population of patients with first-episode psychosis or those identified as being at clinical high risk for developing psychosis.^42^ One limitation of the present study was that the sample included relatively few women. This was not intentional and may reflect the fact that men with psychosis are more likely to use cannabis.^4^ In a recent study of patients with psychosis from the same catchment area, 32% of those who used cannabis were female.^17^ In our study, however, only 5% of participants were women. This difference may partly reflect our recruitment strategy, as a higher proportion of men were approached by the research team. In addition, women were less likely to disclose cannabis use. Women are known to experience more stigma associated with substance use and may have lower levels of trust in healthcare providers.^43^ Second, although the study was framed within a broad psychosis construct, the sample was heavily weighted towards schizophrenia. This limited our ability to draw conclusions about other psychotic disorders and may reduce the generalisability of the findings beyond schizophrenia spectrum conditions. The sample was also predominantly Black and unemployed, reflecting the social disadvantage and structural inequities experienced by many people with psychosis in urban UK settings. These factors were not examined analytically in the present study, and beliefs about cannabis-related harm, illness insight and treatment engagement may be shaped by cultural context, systemic barriers to care and previous negative experiences with services; these aspects also limit the generalisability of our findings.^44^ Other factors that may be relevant, such as age at first use and cannabis potency, were not analysed and could be prioritised in future research. The accuracy of self-reported cannabis use may be lower in clinical than general population samples.^45^ Given that half of our sample expressed a desire to reduce their use, future research should also investigate motivations for wanting to reduce cannabis use and aim to develop interventions to support individuals in reducing or stopping cannabis use.

## Supporting information

10.1192/bjo.2026.12064.sm001 Chesney et al. supplementary material Chesney et al. supplementary material

## Data Availability

Data are available on reasonable request from the corresponding author.

## Supplementary material

The supplementary material is available online at https://doi.org/10.1192/bjo.2026.12064

## Table 1 Long description

The table presents demographic and clinical characteristics of 62 study participants. It includes 15 rows and 3 columns. The columns are labeled Characteristics, and Mean (s.d.; n (%)). The table lists various characteristics such as age, gender, ethnicity, education, employment, marital status, primary diagnosis, duration of illness, psychiatric hospital admissions, antipsychotic use, clozapine treatment, psychosis symptoms, remission, history of violent crime, tobacco, alcohol, and substance use, AUDIT score, cannabis use, preferred type of cannabis, preferred route of administration, DSM-5 cannabis use disorder, and CUDIT-R. Each row provides specific data for these characteristics. For example, the mean age is 42.2 years with a standard deviation of 11.9. The majority of participants are male (95.2%), of Black ethnicity (64.5%), and unemployed (90.3%). The primary diagnosis for most participants is schizophrenia (80.6%). The table also details the frequency and severity of cannabis use, with 87.1% preferring high potency cannabis and all participants smoking cannabis with tobacco. The severity of psychosis symptoms is generally mild, and most participants have severe cannabis use disorder (85.5%).

Navigate back to Table 1.

## Table 2 Long description

A table with 18 rows and 3 columns. The columns are labeled Factor, Odds ratio (95% CI), and P-value. The table lists various factors and their associated odds ratios, confidence intervals, and P-values. Row 1: Age, 0.99 (0.94 to 1.03), 0.44. Row 2: Gender, 1.74 (0.16 to 38.7), 0.66. Row 3: Ethnicity (White), 0.59 (0.19 to 1.81), 0.36. Row 4: Ethnicity (other), 1.48 (0.13 to 33.4), 0.76. Row 5: Cannabis use (grams per day), 2.97 (1.36 to 8.44), 0.018. Row 6: Years of use, 0.99 (0.95 to 1.03), 0.75. Row 7: CUDIT-R score, 1.07 (0.98 to 1.18), 0.15. Row 8: PANSS positive subscale, 1.02 (0.93 to 1.13), 0.64. Row 9: PANSS negative subscale, 0.95 (0.87 to 1.02), 0.19. Row 10: Psychosis insight (PANSS-G12), 0.83 (0.62 to 1.11), 0.22. Row 11: SAS total score, 1.76 (1.30 to 2.53), 0.0007. Row 12: SAS general illness awareness, 1.35 (1.10 to 1.70), 0.007. Row 13: SAS symptom attribution, 1.06 (0.89 to 1.28), 0.52. Row 14: SAS awareness of negative consequences, 1.20 (1.04 to 1.40), 0.014. Row 15: SAS awareness of need for treatment, 1.55 (1.24 to 2.02), 0.0003.

Navigate back to Table 2.
